# Mutagenic Effects of Nanosilver Consumer Products: a new Approach to Physicochemical Properties

**Published:** 2015

**Authors:** Masomeh Heshmati, Sepideh ArbabiBidgoli, Samideh Khoei, Seyed Mahdi Rezayat, Kazem Parivar

**Affiliations:** a *Department of Biology, Science and Research Branch, Islamic Azad University, Tehran, Iran.*; b *Department of Toxicology and Pharmacology, Faculty of Pharmacy, Pharmaceutical Sciences Branch, Islamic Azad University, Tehran, Iran.*; c *Medical Physics Department, School of Medicine, Iran University of Medical Sciences, Tehran, Iran.*; d *Department of Pharmacology, School of Medicine, Tehran University of Medical Sciences, Tehran, Iran.*

## Abstract

Serious concerns have been expressed about potential health risks of Nano silver containing consumer products (AgNPs) therefore regulatory health risk assessment on such nanoparticles has become mandatory for the safe use of AgNPsinbiomedicalproducts with special concerns to the mutagenic potentials. In this study, we examined the inhibitory and mutagenicity effects of AgNPs in three different sizes of three colloidal AgNPs by Minimal Inhibitory concentration (MIC), Minimal Bactericidal Concentration (MBC) and Bacterial Reverse Mutation Assay (Ames test).All samples were characterized by transmission electron microscopy (TEM), X-Ray Diffraction (XRD) and Dynamic Light Scattering (DLS). DLS analysis showed lack of large agglomeration of the AgNPs and TEM results showed the spherical AgNPswith the average sizes of 15, 19.6, 21.8 nms. Furthermore the XRD analysis showed the crystalline samples with a face centered cubic structure of pure silver.AmestestresultsonColloidal silver nanoparticles showed lack of any mutation in TA100, TA98, YG1029S. typhymuriumstrains.In addition colloidal silver nanoparticles reduced the mutation ratesin all three strains in a concentration dependent manner .This finding creates a new issue in the possible antimutagenic effects of colloidal AgNPsas a new pharmaceutical productwhich should be consideredinfuture studiesby focusing onthephysicochemical properties of AgNPs.

## Introduction

For more than a century, Silver nanoparticles (AgNPs) were used as effective antiseptic agents,but in recent decade they have found increasing global concerns because of their strong antibacterial activities in comparison to silver and other antiseptic compounds(1). AgNPs containing products comprising silver nanoparticles, are attracting interest for a range of biomedical and pharmaceutical applications owing to their potent antibacterial activities(2).Moreover it has recently been demonstrated that AgNPhas useful anti-inflammatory effects which may accelerate wound healing (1). The key to its broad-acting and potent antibacterial activity is the multifaceted mechanism by which AgNPacts on microbes. This is utilized in antibacterial coatings onmedicaldevices to reduce the rate of nosocomial infections. Many new synthesis methods have emerged and are being evaluated for AgNPproduction for medical applications (2).

This property has led the global and local markets to many new healthcare and cosmetic products, wound dressings, textiles, contraceptive devices, surgical instruments, bone prostheses, water purification systems, indoor air quality management and other products from AgNPs (3). Increased utilization of nanoparticles in recent years due to significant advances in nanotechnology havean impact on industrial technology with increased risk of human exposure to nanoparticles through occupational and environmental routs (4). 

The antibacterial properties of AgNPs are mainly attributed to their high surface area to volume ratio but this potential may cause their higher reactivity to macromolecules especially DNA reactions, their possible genotoxic effects, mutagen induced health risks propertiesand carcinogenic properties as well (5). According to the possible toxic potentials of AgNPs (6), many studies have revealed the association between genotoxicity of AgNPs and their sizes. Although different size dependent genotoxiceffects from AgNP are reported by different methods in a dose-dependent manner (7), other physiochemical properties including zeta potential, shapes (rods, triangles, spherical particle) and aggregation capacities have been considered for their genotoxic potentials(8)

Out of different genotoxicity assessment methods, the Ames test is known as the most accurate and commonly used procedure to detect genotoxic carcinogens which cause two classes of gene mutation, base pair substitution and small frameshifts (9).Although this test is essential within the current battery of assays required for genotoxicity evaluation and one of key assays recommended by the United Kingdom expert advisory Committee on Mutagenicity (7), its efficiency in genotoxicity assessment of various kinds of nanoparticles has remained inconclusive.This studyaimed to compare themutagenic effects of a wide range of doses ofthree different commercially available colloidal AgNPsin similar sizes on the basis of their physicochemical properties especially by Ames Mutagenicity assay.

## Experimental

*Materials*

*AgNP commercial products*

**Figure 1:**
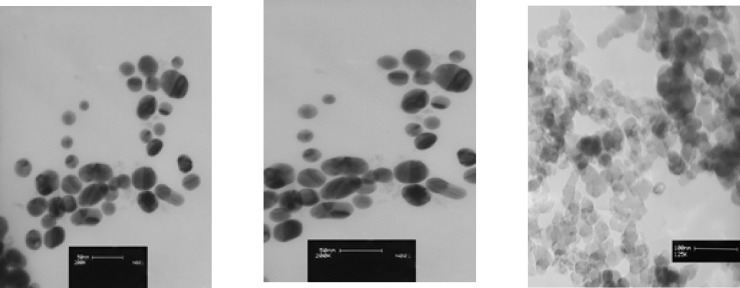
characterization of AgNPs by Transmission Electron Microscopy; A)Representative image of A-AgNP; B) Representative image of B-AgNP; C) Representative image of AgNPs water dispersion

Three types of prevalent commercial products of colloidal silver nanoparticles were used for this study. The first commercial product of AgNPswas a yellowish-brown product, purchased from a local manufacturer in Tehran and coded as A-AgNP. According to the information provided by manufacturer, it was a water-based colloid which contained 4000 mg/ml spherical uncoated AgNP. The size and zeta potential of this sample was provided by TEM and DLS methodrespectively (Figure 1).

The second commercial AgNPs was donated by another local manufacturer in Isfahan and coded as B-AgNP. According to the information provided by manufacturer, it was water-based plant coated colloid contained 187.5 mg/ml of spherical silver nanoparticles. The size and zeta potential of this sample was provided by TEM and DLS method respectively(Figure 1).

The third colloidal silver nanoparticle was purchased from US Research nanomaterials co, Ltd (TX, USA).This product was a water based colloidal AgNPcontaining 1000 mg/ml spherical silver nanoparticles with 99.9% purity according to the information provided by the supplier. The size and zeta potential of this sample was provided by TEM and DLS methodrespectively (Figure 1).

*Characterization of nanoparticles*

The concentration of samples was determined by Atomic Absorption Spectroscopy (AAS). AgNPs were homogeneously dispersed in sterile distilled water by sonication for 30 minutes and filtered. Transmission electron microscopy (TEM) was used to estimate the size, shape and composition of the AgNPs. The distribution of the AgNPs, hydrodynamic size and zeta potential was evaluated using Dynamic light Scattering (model ZEN3600; Molvern instrument Ltd, Tokyo, Japan). The X-ray diffraction pattern was performed in order to characterize nanoparticle structure. Experimental methods were used to optimize the concentrations of each serial diluted samples and to assure the quality of provided data. All experiments were repeated at least three times to confirm the accuracy and reproducibility of this method. Standard gold nanoparticles with pre-determined size were used to validate the instrument. Both above parameters (size and zeta potential) were measured at least three times for each sample. The data were calculated as the average size or zeta potential of AgNPs.

*Salmonella strains*

The Salmonella Typhymurim strains used in this study were TA100, TA98 and YG1029 a bacterial O-acetyltransferase-overproducing strain of TA100(10).The YG1029 was cloned and its activity was first described and established by T Nohmi and colleagues(11) who provided all strains as gift, from Biological Safety Research Center co, Ltd in Tokyo-Japan (2012).

*MIC and MBC levels*

The Minimal Inhibitory Concentration (MIC) value is the lowest concentration of AgNPs which prevents bacterial growths. Appropriate concentrations of each AgNPs samples were prepared according to their working manual,using muellerhintonbrothin the range of 31.2-0.015 µg/ml with serial two fold dilutions of 1, 1/2, 1.4, 1/8, 1/16, 1/32, 1/64, 1/32, 1/64, 1/128, 1/256, 1/512, 1/1024 inoculate 1×10^8^ colony forming units (CFU)/ml of tester strains. After 24h from incubation at 37ºC,MIC values for each strain were determined by choosing the lowest concentrations in which no growth occurs. Additionally, the minimal bactericidal concentration (MBC) values were determined by sub -culturing the content of each tube without any growth, on the Mueller Hinton agarmedium and looking for any bacterial growth (1). The MIC value was considered as the concentration with 99% bacterial growth inhibition and the MBC value was considered as the concentration with 100% of the inhibitory properties in comparison to the negative control.

*Bacterial Reverse Mutation Assay*

The pre-incubation Ames assay was performed according to the method of OECD 471(13) .Before starting the main experiments, the mentioned strains were checked for their genetic integrity by histidine/biotin dependence, histidine dependence, biotin dependence, rfa marker(crystal violet) and the presence of plasmid pKM101 (Ampicillin resistance) tests (14; 15; 16).During all these preliminary experiments, the strains were grown overnight in nutrient brothfor 16-18h in incubator at 37ºC with a density of 1 - 2 × 10^8^ (CFU) ml in presence of 25 µg/ml Ampicilin for TA98, TA100 and 10 µg/ml Tetracycline for YG1029). The top agar was supplemented with histidine/biotin and prepared by dissolving 0.6g of agar-agarand 0.6g NaCl in 100 ml distilled water. A sterilized aqueous solution of L-histidine and D-biotin (0.5 mM/L) was added to top agar medium immediately before applications (2; 3). In the next step, 100 µl of overnight cultured bacteria with concentration of 1 - 2 × 10^8^ CFU/ml were incubated at 37ºC for 45 min in a sterile glass tube containing 500 µlit sodium phosphate buffer (0.1 M, pH7.4) with the different concentrations of the colloidal AgNPs. The concentration of AgNPs used in this study was 3.12-0.0015 µg/plate. After incubation, 2 ml of Top agar supplemented with histidine/biotin (kept in 45ºC water bath) and added to the mixture and mixed for 3 s using a vortex mixer, then poured on a plate of minimal glucose agar media (16). Plates were incubated for 48-72 h at 37ºC and the revertant colonies were counted. Three equal plates were used for each concentration and each experiment was repeated three times to get maximum accuracy and reproducibility for this sensitive method (17).

*Data analysis*

For the Ames mutagenicity assay, positive responses required a dose-related increase in the number of revertant colonies/plate for each of strains and sodium azide at5 µg/ml concentration (Sigma Aldrich, cat number: 438456) was used as positive control. Negative response was defined as no concentration related increase in the number of revertant colonies and distilled water was used as negative control in this study. Also, a positive controlwas defined as an agent with defined concentration which can double the number of revertantcolonies/plate in comparison to the vehicle control(17).The Mutagenic Index (M.I) was described for each assay and calculated as the ratio between number of histidinerevertants induced per plate of the test sample and spontaneous revertants of the negative control(18).

Statistical analysis was performed using IBM SPSS statistics software version 20 and one-way ANOVA (LSD) was used to compare the colony counts of each plate in different concentrations and groups.Multifactorial analysis was carried out by considering the concentration as the main factor and p-values 0.05 was considered as statistically significant changes.

## Results and Discussion

*AgNPs characterization*

As described above, three samples of commercial AgNPs were characterized by TEM, DLS and XRD methods (Table 1). The average particle sizeof aqueous colloidal silver nanoparticles A-AgNP, B-AgNP and C-AgNPwas19.6, 21.8 and 15 nm respectively. Shapes of AgNPs with 21.8 and 15 nm were spherical and different shapes were observed in AgNPs with 19.6 nm (Figure 1). All nanoparticle suspensions were stable and monodispersed with normal pH. The XRD showed crystalline pattern in all three samples .All samples had a face centered cubic structure of pure silver (Table 1)

**Table 1:** Physicochemical characteristics ofAgNPs

Samples	Concentration^1^ (µg/ml)	Shape^2^	TEM size(nm^2^)	Hydrodynamic size(nm^3)^	Zeta potential mV(pH)^2^	Coting Style	Specific Gravity(cP)	pH	PDI
A-AgNP	4000	different	19.6	83.7	-12	uncoated	1.14	2.5	0.608
B-AgNP	187.5	spherical	21.8	52.56	-24.6	Plant coated	0.8872	4	0.516
C-AgNP	1000	spherical	15	62.4	-11.29	uncoated	ND^4^	7	ND

1 Determined by Atomic Absorption Spectroscopy (AAS)

2 Determined by Transmission Electron Microscopy (TEM)

3 Determined by Dynamic Light Scattering (DLS)

4 Not determined

*MIC and MBC determination by broth serial macrodilution method*

The inhibitory effects of different concentrations of AgNPs on three Salmonella strains were examined and described in Table 2-4. Most of our experiments showed clearly that MIC and MBC levels were equal in all three samples. MIC and MBC of A-AgNP (19.6 nm) in TA100, TA98 and YG1029 were 3.9, 3.9, 7.81 µg/ml respectively. MIC and MBC of B-AgNP (21.8 nm) in TA100, TA98 and YG1029 were also 3.9, 7.81, 15.6 µg/ml respectively. MIC and MBC of C-AgNP (15 nm) was equal to 3.9 µg/ml in TA98 but in TA100 and YG1029 were 3.9, 1.9 and 7.81, 3.9 µg/ml respectively.

*Mutagenic potentials of AgNPs by Ames test*

As described above, bacterial mutagenicity was assessed in S.typhimurium tester strain TA98 for detection of frameshift mutation, TA100 for measurement of base pair substitution and YG1029, that was derived from TA100, with the enzymatic activity of O-acetyltransferase for its point mutation capacities via metabolic activation. Details are described below:

*Mutagenic Potentials of A-AgNPs*

Significant increase in the number of revertant colonies was detected in positive control plates and the number compared with negative control (< 0.001).The Mutagenicity Index (MI) was recorded as 9.75 ± 0.16 for TA98, 4.8 ± 0.009 for TA 100 and 13.1 ± 0.35 for YG1029.This observation indicated that the test system is sensitive and specific to the mutagenic potentials of sodium azide in all three tester strains (Table 2).

**Table 2:** Mutagenicity ofA-AgNPs inS. Typhimurium tester strains (TA98, TA100, YG1029)^1^.

**Mutagenic Index** **(** **SEM ± Mean** **)**			**Number of revertant colonies/Plate** **(** **SEM ± Mean** **)**			**Dose** **µg/plate)** **)**
MI_YG1029_	MI _TA100_	MI _TA98_	YG1029	TA100	TA98	
1.00±0.00*	1.00±0.00	1.00±0.00	55.3±2.6	197±2.08	95.6±2.9	0.00
0.02 ± 0.76*	0.78 ± 0.03 *	0.78 ± 0.03 *	55.3 ± 1.2	153 ± 1.7	71.3 ± 1.45	0.0015
0.68±0.02*	0.86±0.01*	0.79±0.03*	37.3±1.2	170±1.2	±1.769.6	0.003
0.84±0.03*	0.77±0.006*	0.75±0.02*	46.3±1.4	153±1.73	2.03 ±73.3	0.006
0.87±0.05*	0.79±0.008*	0.58±0.02*^3^	48.3±0.88	156.3 ± 1.73	2.1 54.3±	0.012
0.9±0.05*	0.82±0.004*	1.00±0.03	49.6±0.66	162.3±1.85	96.3±2.02	0.024
0.64±0.02*	0.83±0.002*	0.85±0.01*	35.6±0.88	164.3±1.3	79±2.6	0.048
0.73±0.01*	0.96±0.003^4^	o.8±0.01*	40.6±1.2	190.3±1.4	74.3±2.02	0.097
0.76±0.05*	0.87±0.003	0.84±0.08*	42.3±0.88	172.6±3.2	72.3±1.6	0.195
0.84±0.04*	0.89±0.01	0.98±0.03	46.6±1.2	176.3±1.6	91±2.08	0.39
0.67±0.04*	ND	ND	37±1.1	T	T	0.78
ND			T			1.56
						3.12
13.1±0.35	4.8±0.009	9.75±0.16	726±13.1	948±10.7	926.6±2.08	Positive control

1 Note: Above data represents the mean number of revertant colonies ± SEM from three independent experiments, each repeated three times.

2 Note: T denotes the toxic concentration of A-AgNP.Doses higherthan T could not be evaluated against; the positive control (sodiumazidwith 5 µg/ml) and control negative (distilled water) and described as Not Determined (ND).

3 Highest inhibitory level was 0.58 ± 0.02.

* p < 0.001, p < 0.05vs control.

The number of colonies and the MIs were calculated for 12 dilutions of A-AgNP .Concentrations upper than 0.39 µg/platewere considered toxic for TA98 and concentrations upper than 0.78 µg/platewere considered toxic for TA100 and YG1029 (Table 2).

Out of 12 evaluated concentrations of A-AgNP, 8 concentrations (0.0015-0.0195 µg/plate) showed lack of mutagenic potentials when compared with positive and negative controls.Significant lower colony numbers and MIs in above dilutions in comparison to the colony numbers in TA98 (95.6 ± 2.9), TA100 (197 ± 2.08) and YG1029 (55.3 ± 2.6) showed inhibitory effects of Nanosilver with determined physicochemical properties in these strains .Moreover YG1029 showed more sensitivity to higher concentrations of A-AgNP in this study (Table 2).

*Mutagenic Potentials of B-AgNPs*

Significant increase in the number of revertant colonies was detected in positive control plates when the number compared with negative control (< 0.001).The Mutagenicity Index (MI) was recorded as 9.2 ± 0.13for TA98, 4.5 ± 0.14 for TA 100 and 7.5 ± 0.19 for YG1029.This observation indicated that the test system is sensitive and specific to the mutagenic potentials of sodium azide in all three tester strains (Table 3).The number of colonies and the MIs were calculated for 12 dilutions of A-AgNP.Concentrations upper than 0.39 µg/plate were considered toxic for TA98 and TA100 and concentrations upper than 1.56 µg/platewas considered toxic for YG1029 (Table 3).

**Table 3:** Mutagenicity of B-AgNP in S. Typhimurium tester strains (TA98, TA100.YG1029)^1^.

Mutagenicity Index (SEM±Mean)			Number of revertant colonies/ Plate (SEM±Mean)			Dose µg/plate))
MI_YG1029_	MI _TA100_	MI _TA98_	YG1029	TA100	TA98	
1.00±0.00	1.00±0.00	1.00±0.00	72±1.4	196.6±8.81	100±0.57	0.00
*0.76±0.02	0.78±0.03*	0.78±0.03*	55.3±1.2	153±1.7	71.3±1.45	0.0015
0.72±0.01*	0.88±0.04*	0.88±0.04*	52.3±1.2	173.3±2.02	61.3±1.45	0.003
0.68±0.01*	0.73±0.04*	0.73±0.04*	50±1.5	143.3±2.02	64.3±2.1	0.006
0.79±0.06*	0.98±0.03*	0.98±0.03*	57±1.2	192.3±1.8	73±1.15	0.012
0.86±0.01*	0.82±0.03*	0.82±0.03*	62±2.1	161.3±0.8	82±1.52	0.024
0.79±0.02*	0.89±0.03*	0.89±0.03*	58±2.6	175.3±1.4	70.6±1.2	0.048
0.65±0.02*	0.97±0.03*	0.97±0.03*	48±2.5	192±1.5	84±1.73	0.097
0.82±0.02*	0.92±0.04*	0.92±0.04*	59±2.9	180±1.4	94.3±2.4	0.195
0.84±0.03*	ND	ND	61±3.8	T	T	0.39
0.85±0.01*			62±1.5			0.78
ND			T			1.56
						3.12
7.5±0.19	4.5±0.14	9.2±0.13	580±20.8	893±12	926.6±14.5	Positive control

1 Note: Above data represents the mean number of revertant colonies ± SEM from three independent experiments, each repeated three times.

2 Note: T denotes the toxic concentration of A-AgNP. Doses higher than T could not be evaluated against; the positive control (sodium azidwith 5 µg/ml) and control negative (distilled water) and described as Not Determined (ND).

* p < 0.001, p < 0.05vs control.

Out of 12 evaluated concentrations of A- AgNP, 8 concentrations (0.0015-0.195 µg/plate) showed lack of mutagenic potentials when compared with positive and negative controls .Significant lower colony numbers and MIs in above dilutions in comparison to the colony numbers in TA98 (100 ± 0.57), TA196.6 (197 ± 8.81) and YG1029 (72 ± 1.4) showed inhibitory effects of Nanosilver with determined physicochemical properties in these strains .Moreover YG1029 showed more sensitivity to higher concentrations(0.39 and 0.78 µg/plate) of A-AgNP in this study (Table 3).

*Mutagenic Potentials of C-AgNPs*

Significant increase in the number of revertant colonies was detected in positive control plates when the number compared with negative control (< 0.001).The Mutagenicity Index (MI) was recorded as 9.8 ± 0.28 for TA98, 4.8 ± 0.05 for TA 100 and 8.3 ± 0.55 for YG1029.

This observation indicated that the test system is sensitive and specific to the mutagenic potentials of sodium azide in all three tester strains (Table 4).The number of colonies and the MIs were calculated for 12 dilutions of C-AgNP .Concentrations upper than 0.195 µg/plate were considered toxic for TA98 and TA100 and concentrations upper than 0.39 µg/plate was considered toxic for YG1029 (Table 4).

**Table 4.** Mutagenicity of C-AgNPs in S.Typhimurium tester strains (TA98, TA100, YG1029)^1^.

**Mutagenicity Index** **(** **SEM ± Mean** **)**				**Number ofrevertant colonies/Plate** **(** **SEM ± Mean** **)**				**Dose** **µg/plate)** **)**	
MI_YG1029_	MI _TA100_	MI _TA98_		YG1029	TA100	TA98			
1.00±0.00	1.00±0.00	1.00±0.00		69.9±2.4	190.3±2.6	197±3.2		0.00	
0.64±0.05*	0.9±0.01*	0.52±0.01*		45±2.8	174.3±2.3	95.6±2.3		0.0015	
0.75±0.01*	0.8±0.01*	0.66±0.004*		52.6±1.4	160±0.5	50.6±1.7		0.003	
0.97±0.02	0.7±0.01*	0.85±0.02*		67.6±1.2	143.3±0.3	63.6±0.8		0.006	
0.87±0.03	0.7±0.001*	0.78±0.01*		61.3±4.09	139.6±2.02	81.3±1.00		0.012	
0.71±0.06*	0.7±0.007*	0.94±0.01		49.3±2.72	151.6±0.6	75±1.2		0.024	
0.93±0.017	0.8±0.01*	0.87±0.01*		65.3±3.17	170±0.57	90.6±1.4		0.048	
0.99±0.06	0.7±0.01*	0.97±0.02		69.3±2.02	135±2.6	83.3±1.00		0.097	
0.85±0.02	0.7±0.01*	0.92±0.04		59.3±0.88	143±1.7	93±0.8		0.195	
0.70±0.05*	ND	ND		49±2.3	T	T		0.39	
ND				T				0.78	
								1.56	
								3.12	
8.3±0.55	4.8±0.05	9.8±0.28		580±25.1	926.6±20.8	943.3±17.6		Positive control	

1 Note: Above data represents the mean number of revertant colonies ± SEM from three independent experiments, each repeated three times.

2 Note:T denotes the toxic concentration of A-AgNP. Doses higher than T could not be evaluated against; the positive control (sodium azidwith 5 µg/ml) and control negative (distilled water) and described as Not Determined (ND).

* p < 0.001, p < 0.05vs control.

Out of 12 evaluated concentrations of A- AgNP, 8 concentrations (0.0015-0.195 µg/plate) showed lack of mutagenic potentials when compared with positive and negative controls. Significant lower colony numbers and MIs in above dilutions in comparison to the colony numbers in TA98 (197 ± 3.2), TA100 (190.3 ± 2.6) and YG1029 (69.9 ± 2.4) showed inhibitory effects of Nanosilvercolloidal solution with determined physicochemical properties in these strains.Moreover YG1029 showed more sensitivity to a higher concentrations 0.39 µg/plateof C-AgNP in this study (Table 4).

Our research focused on genotoxic effects of silvernanoparticles (AgNPs), which can be utilized in biomedical research, pharmaceutical products and environmental cleaning applications(19). Despite the widespread use of AgNPs in a wide range of biomedical products as a new group of health products,there are numerous knowledge gaps regarding their possible toxic potentials especially their mutagenic effects in human (8), (7). We described previously systemic toxic effects of AgNPs through dermal application in animal models (4), (5), (6) but limited studies on genotoxic effects ofAgNPs with dermal exposure has motivated us to continue our toxicity evaluations in this new area and this new bacterial settings for nanomaterials.

**Figure 2:**
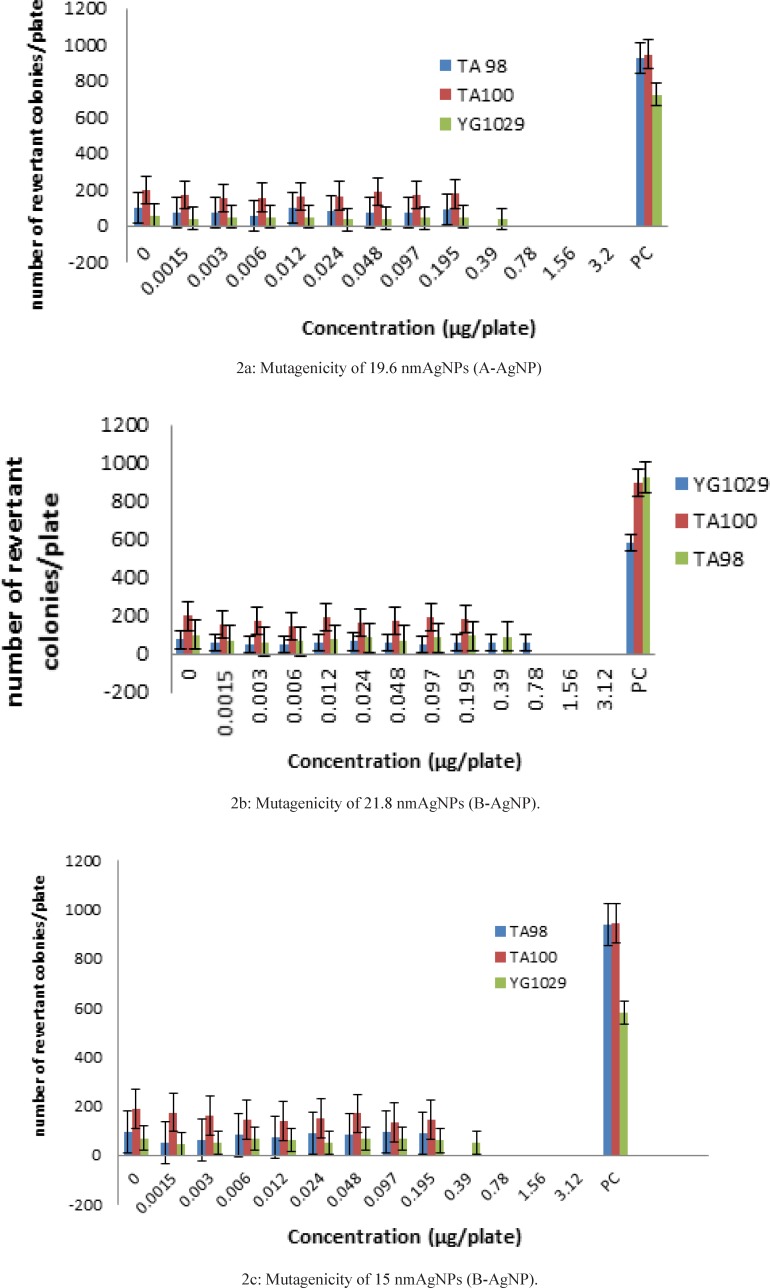
Comparison of mutagenic effects of AgNPs with positive controlinS. typhimuriumtester strains (TA98, TA100. YG1029).

We defined three distinctive statistical groups as ‘‘A’’, ‘‘B’’, and ‘‘C’’ with 15-22 nm size and compared each one with negative control (Table 2-4) and positive control (Figure 2:2a, 2b, 2c). The rational of this size selection was based on the results of one recent study which had introduced 20 nm AgNPsmuch more toxic than larger sizes due to their higher dissolution rate and larger surface area to volume ratio (7). Revertant colonies didn’t induced by the samples in a wide range of nontoxic concentrations but low AgNP concentrations showed significant inhibitory effects on TA98, TA100 and YG1024. In accordance to mentioned results, larger size AgNPs with 40-50 nm diameter (8) and lower sizes of AgNP with 5 nm diameter (9) could stop the growth of TA100 and TA98, in a dose-dependent pattern but as a novel issue lower levels of revertant colonies even lower than the negative control responded properly. Although this preliminary finding suggests again the same result as before in a new range size, the clear and significant microbial inhibitory effects of all three samples (p < 0.001) suggest their possible antimutagenic effects of AgNPs with certain physicochemical properties which should be considered for future experiments on certain mutagenic agents.

Other than the impact of size in mutagenic capacity,we considered the direct role of concentration and the indirect roles of shape, coating,zeta potential, PDI, pH and viscosity in this classic bioassay (3). The role of nanoparticle shapes e.g. rods, tatriangles and spherical particles (10) on bacterial toxicity has been described before in concentrations lower than MIC and MBC values (11).The shape of nanoparticles may modify the toxicity of AgNPs by changing the extents of Ag^+ ^ions (11)but other than the role of shape,impact of coating was considered for gram negative bacterium pseudomonas putida and results showed no simple clear-cut relation between the toxicity of the different particles and their shapes and coatings.Actually the ion release kinetics could be considered as one the major factors for genotoxic effects of AgNPs, an issue which was not determined by us (12), (13).

Our result showed weak effects of physicochemical properties on mutagenicity potentials of AgNPS as well as no simple clear-cut relation between coating and zeta potential of materials.There are several explanations for these negative ames results. Some studies showed that the negative results may be due to the inability of AgNPs to penetrate through the bacteria cell wall and lack of bacterial cells to perform endocytosis and incorporate them (8; 9; 14) but the underlying factor was the agglomeration of AgNPs which makes the particles too large to transport through the pores in bacterial cell wall. This lack of uptake could potentially lead to false negative results.

Colloidal silver nanoparticles did not induce mutations in TA100, TA98, YG1029 S.typhymuriumstrains, so far they reduced the rate of mutaions in all three strains in a concentration dependent manner, a new issue which should be analyzed in next studies to assess their antimutagenic potentials. Moreover these data suggest the technical weakness of Ames test to evaluate the mutagenicity of the AgNPs.It seems that more genotoxic methods e.g. micronucleous assay, comet assay,chromosome aberration assay are necessary to assess the nanoparticle genotoxicity and their distinctive characteristics including size, shape, zeta potential, solubility, surface area.
